# ASO Author Reflections: Loss of Intestinal Integrity During Oncological Surgery and Postoperative Complications: A Complex Relationship

**DOI:** 10.1245/s10434-024-14980-z

**Published:** 2024-01-22

**Authors:** Sharon Hendriks, Barbara L. van Leeuwen, Jacco J. De Haan

**Affiliations:** 1 Department of Surgery, University of Groningen, University Medical Center Groningen, Groningen, The Netherlands; 2 Department of Medical Oncology, University of Groningen, University Medical Center Groningen, Groningen, The Netherlands

## Past

Compromised intestinal integrity due to surgery has been thought to negatively impact surgical outcomes. Splanchnic hypoperfusion, which may be caused by vasodilatory anesthetic drugs, the inflammatory response, or acute blood loss, is considered an important contributor to perioperative loss of intestinal integrity.^1^ Loss of intestinal integrity and its association with perioperative blood pressure and postoperative complications, however, has not been studied yet in patients undergoing oncological surgery.

## Present

In this exploratory study, in 297 patients undergoing surgery for solid tumors, urine concentrations of intestinal fatty acid binding protein (I-FABP) were determined preoperatively (T0) and at wound closure (T1).^2^ An increase of I-FABP in urine reflects loss of intestinal integrity.^3^ This study is the first to show that intestinal integrity is compromised during oncological surgery, as indicated by an increase of median I-FABP [from 80.0 pg/mL (IQR 38.0–142.0) at T0 to 115 pg/mL (IQR 48.0–198.0) at T1 (*p* < 0.05)]. Age and length of anesthesia were related to stronger I-FABP increases. Remarkably, longer periods of mean arterial blood pressures (MAP) below 65 mmHg were not related to an increase of I-FABP. Patients experiencing complications, in particular inflammation, showed a greater postoperative I-FABP increase compared with patients without complications.^2^ In conclusion, this study shows that intestinal integrity is compromised during oncological surgery and is related to postoperative complications. The relation between intestinal integrity and hypoperfusion remains unclear.

## Future

Future studies should standardize I-FABP measurement to allow for better comparison between studies and to gain more insight into the relation between loss of intestinal integrity and postoperative complications. To determine the importance of mean arterial pressure and splanchnic perfusion in this context, individual threshold blood pressure cut-offs based on patients’ normal blood pressure should be determined. Next, patients’ intestinal vascular status with concomitant measurements of mesenteric perfusion should be assessed preoperatively.
